# A Case of May-Thurner Syndrome

**DOI:** 10.7759/cureus.10489

**Published:** 2020-09-16

**Authors:** Harini Lakshman, Mahmoud Barbarawi, Pal Satyajit Singh Athwal, Michele Obeid, Ghassan Bachuwa

**Affiliations:** 1 Internal Medicine, Michigan State University at Hurley Medical Center, Flint, USA; 2 Internal Medicine, Saraswathi Institute of Medical Sciences, Hapur, IND

**Keywords:** iliac vein thrombosis, may-thurner syndrome

## Abstract

May-Thurner syndrome, which is also known as iliac vein compression syndrome, is caused when an anatomical variant of the left common iliac vein with a lateral or anterior spur is compressed by the right iliac artery, resulting in thrombosis of the vein. It can present as left deep vein thrombosis which can lead to pulmonary embolism or chronic changes of venous insufficiency in the left lower limb. We report a 27-year-old female with pain abdomen, who was diagnosed to have May-Thurner syndrome.

## Introduction

May-Thurner syndrome was first described by May and Thurner in 1957, in which a spur-like projection in the left common iliac vein is compressed by the right common iliac artery [1]. This compression is reportedly associated with intimal hyperplasia and venous stasis, which can be a potential cause of thrombosis [2]. Autopsy data reported this "spur" in more than 20% of the population but is rarely considered in the diagnosis of deep vein thrombosis (DVT) involving iliac veins [1]. The right common iliac artery compresses the left iliac vein against the fifth lumbar vertebrae leading to spur formation and venous thrombosis due to impaired venous return and fulfilling one of Virchow's triad, which is stasis, hypercoagulability state, and endothelial injury. It presents as unilateral left DVT leading to skin changes and chronic left lower extremity pain. Diagnosis is based on clinical presentation, and Doppler can be used; however, CT, MRI, and conventional venography are more helpful in detecting location of iliac vein thrombosis and compression. Conventional venography remains the gold standard for diagnosis.

Treatment modalities involve thrombolysis and stenting, both of these are considered as first line. In case of stent placement lifelong anticoagulation is indicated. Mechanical or catheter-based thrombolysis with tissue plasminogen activator (tPA) can also be used. The patient should be assessed for development of any complication or venous thrombosis. We report a case of this abnormal anatomic variation leading to left iliac vein thrombosis presented with atypical symptom of abdominal pain. The patient was adequately treated before development of any fatal complication.

## Case presentation

**Figure 1:**
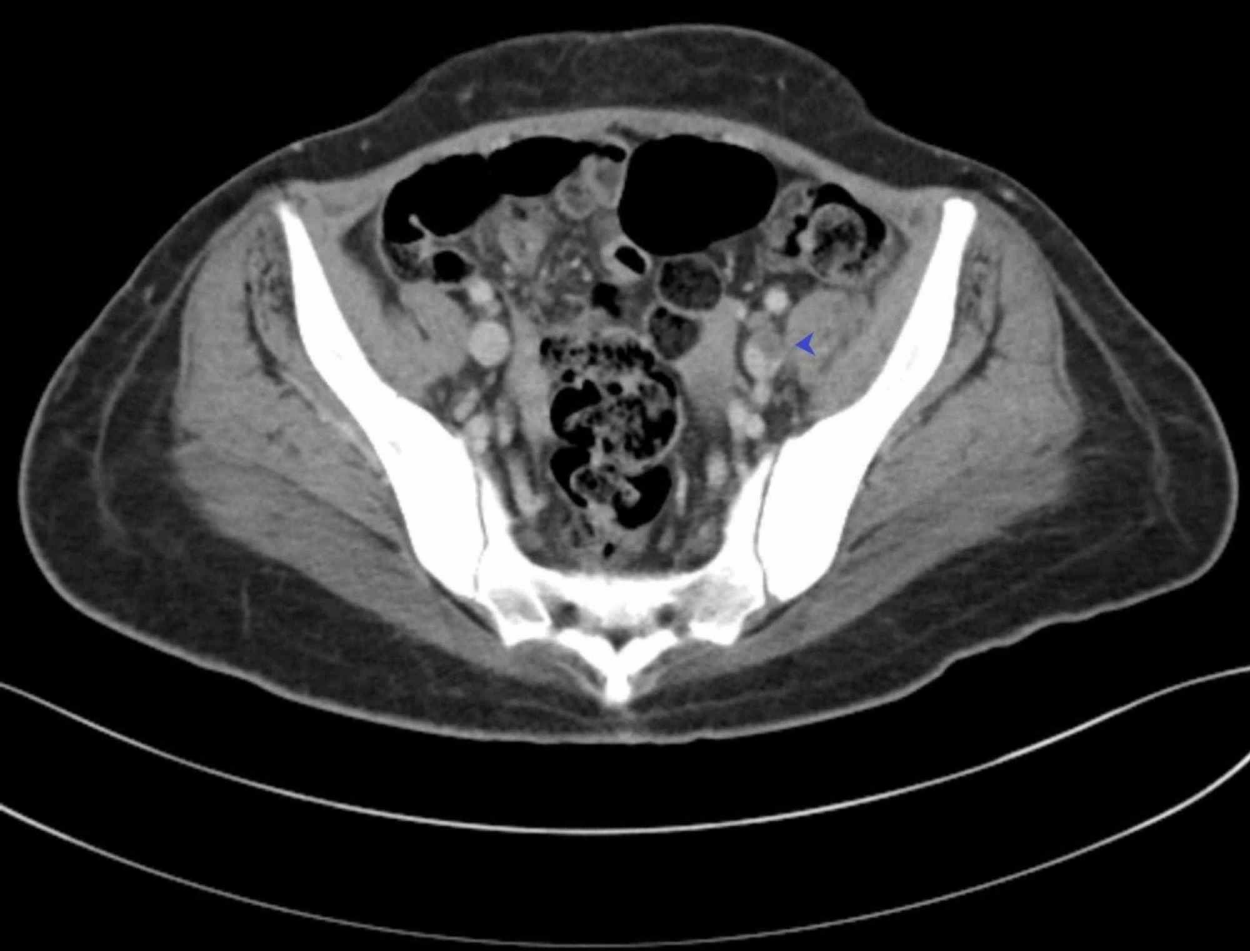
CT scan showing iliac vein thrombosis

**Figure 2:**
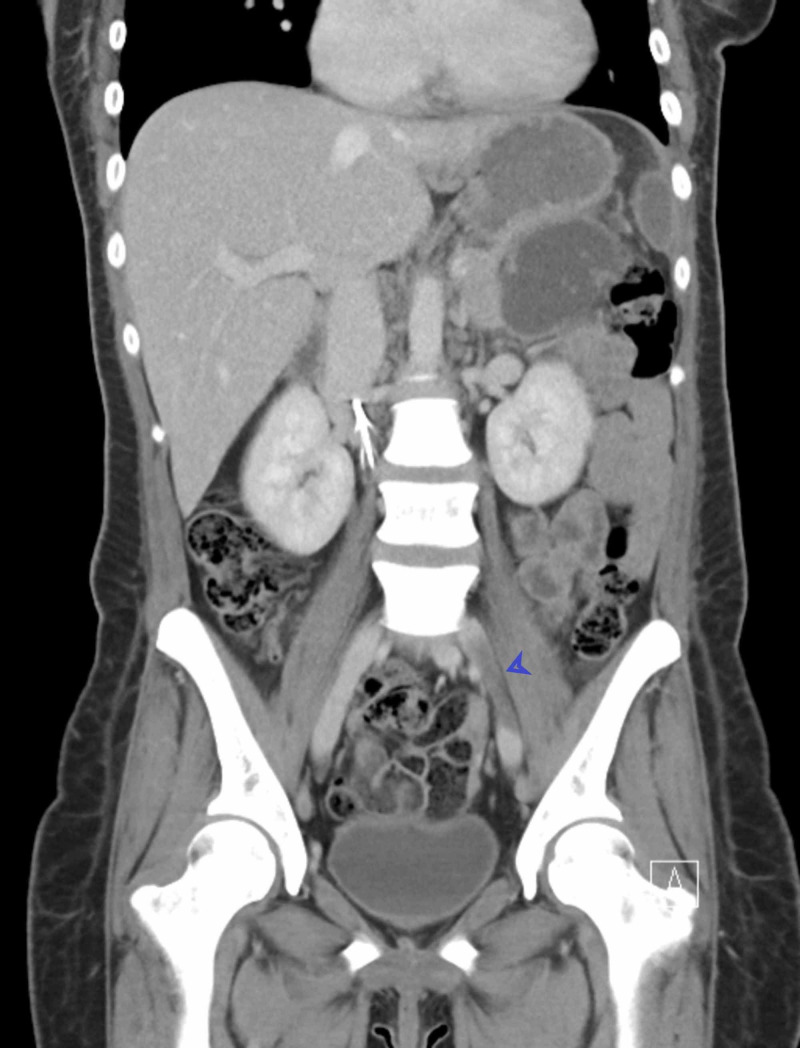
Extension of thrombosis can be seen in CT scan

A 27-year-old African American female, who has a history of seven pack-years of tobacco use and Graves’ disease, was admitted for left lower abdominal pain. Abdominal CT was done and showed a thrombus within the left common iliac vein extending into the proximal inferior vena cava (IVC) and terminating below the level of the renal veins (Figures 1, 2).

The patient did not have any past medical history of vein thrombosis, immobility, trauma, or malignancy. An extensive workup was negative for any hematologic thrombophilic disease. Coagulation profile was done to rule out hypercoagulable states like factor V deficiency, protein C and S deficiency, and antithrombin deficiency. On the abdominal CT scan, the left common iliac vein was compressed between the lumbar spine and the right common iliac artery, and this was the reason behind the DVT formation (May-Thurner syndrome). A venogram was done, and the left iliac vein was clearly compressed between the vertebral column and the right iliac artery. The diagnosis of May-Thurner syndrome was confirmed, and the patient underwent thrombectomy and IVC filter placement. The patient was subsequently started on anticoagulation.

## Discussion

May-Thurner syndrome is reported only in very few patients [3]. This may be under-reported given the lower extremity DVT is more common on the left side as first observed by Virchow [4]. Pathophysiology involves abnormal anatomic variation of the left iliac vein, which lead to compression against fifth lumbar vertebrae by the right common iliac artery. Compression of vein leads to spur formation and along with venous stasis leads to venous thrombosis. Pulsating compression is believed to be responsible for intimal fibrosis and elastin deposition in the iliac vein.

The majority of left lower extremity DVT is in young female patients of age between 25 and 50 years, and the diagnosis is often attributed to some other causes (Table 1); therefore, May-Thurner syndrome is often missed [3,5].

**Table 1:** Some of the common causes of deep vein thrombosis

Etiological factors
Recent surgery
Pregnancy
Oral contraceptives
Inherited hypercoagulable states
Immobilization

Failure to diagnose patients with May-Thurner syndrome will lead to the recurrence of DVT, with additional complications like chronic venous stasis, iliac vein rupture, or pulmonary embolism [6]. This patient was 27 years old when diagnosed, and features like unilateral left DVT were absent. Other causes of thrombophilia should always be ruled out as was done in this case.

If May-Thurner syndrome is suspected, the diagnosis is usually made by MRI, venography, or IV ultrasound [7]. In this case as the patient presented with abdominal pain, CT was performed that showed left iliac vein thrombosis. Once the diagnosis is confirmed, the mainstay of therapy is a combination of thrombolytics and percutaneous mechanical thrombectomy. It has also been suggested that an IVC filter be placed prior to intervention to prevent further embolization during lytic therapy [5,7]. Systemic anticoagulation therapy is recommended for at least six months after the surgical intervention [5].

## Conclusions

May-Thurner syndrome is thrombosis of the left iliac vein due to compression by the right iliac artery against the fifth lumbar vertebrae. It should be considered in differential diagnosis of abdominal pain or patient with unilateral DVT. Diagnosis is crucial to initiate appropriate treatment. It presents as left DVT and chronic changes over left lower limb due to venous stasis. This case presented with abdominal pain, and classical signs like DVT and pain in the lower extremities were absent; in such a scenario, diagnosis can be missed which can lead to fatal complications like pulmonary embolism or recurrent thrombosis. The patient was appropriately thrombolyzed after diagnosed on CT scan. The goal of this case report is to familiarize physicians of atypical presentation of such a case. 
